# Signs and symptoms of covid − 19 in patients with a history of coronary artery bypass grafting surgery

**DOI:** 10.1186/s12879-024-09090-w

**Published:** 2024-02-22

**Authors:** Yasaman Rezvani Emamzadehashemi, Khashayar Rezvani Emamzadehashemi, Atefeh Ghanbari Khanghah, Ezzat Paryad, Marzieh Jahani Sayad Noveiri

**Affiliations:** 1 Guilan University of medical sciences, Rasht, Iran; 2 Department of neurosurgery, Faculty of Medicine, Imam Khomeini Hospital, Urmia University of Medical Sciences, Urmia, Iran; 3 Social Determinants of Health Research Center (SDHRC), Department of Nursing, Guilan University of Medical Sciences, Rasht, Iran; 4 Department of Nursing, GI Cancer Screening and Prevention Research Center, Guilan University of Medical Sciences, Rasht, Iran; 5 Department of Medical Surgery, School of Nursing and Midwifery, Guilan University of Medical Sciences, Rasht, Iran

**Keywords:** Coronary artery bypass, COVID-19, SARS‐CoV‐2, CABG

## Abstract

**Background:**

People who have coronary artery disease are more likely to develop signs and symptoms of COVID-19 due to their special circumstances. Coronary artery bypass grafting surgery (CABG)does not cure the disease but reduces the signs and symptoms, therefore, there is a possibility of severe complications of Covid-19 after it.

**Materials and methods:**

This study is a descriptive and cross-sectional study conducted from June to July 2020 on 200 patients who underwent CABG from February 2018 to February 2020. The instrument consisted of socio-demographic variables and COVID’s signs and symptoms checklist. Data were collected by census method by telephone. Data were analyzed using descriptive statistics, Fisher’s exact test, Mann Whitney U test, and logistic regression model.

**Results:**

The results showed that the majority of the samples were male (67%). The mean age of them was 62.02 ± 9.06 years and 10% of the m had signs and symptoms of Covid 19. Having the symptoms of COVID-19 is significant in terms of the variables of decreased sense of smell (*p* < 0.002), decreased sense of taste (*p* < 0.002), and home quarantine (*p* < 0.01). The logistic regression model showed decreased sense of taste (OR = 6.071, CI95%: 1.621–29.984, *p* < 0.009) and non-compliance with home quarantine (OR = 0.061, CI95%: 0.005–0.741, *p* < 0.028) were the related variables to signs and symptoms of Covid 19.

**Conclusion:**

The results did not indicate the frequency of COVID signs and symptoms among people with a history of Coronary artery bypass grafting surgery more than healthy people in the Iranian community. Extensive studies are suggested in this regard.

## Background

Coronary artery bypass graft surgery (CABG) is performed to reduce the signs and symptoms of coronary artery disease in cardiac surgery centers [1]. Despite the effective effects of this surgery on slowing the progression of signs and symptoms of coronary artery disease, the use of a heart and lung pump, which in most cases is necessary for this surgery, can be associated with complications for the patient [2]. One of the side effects of using a cardiopulmonary bypass pump is its effects on the immune system [3]. Using this pump can cause general inflammatory responses in the body [4]. The immune system’s response to the use of a cardiopulmonary pump can sometimes take a long time [5]. Despite careful care, unfortunately, respiratory dysfunction after heart surgery is also a complication that is very common in many patients [6].

Unfortunately, the world today is caught up in the epidemic of the Covid 19 virus [7, 8]. About 80% of patients recover spontaneously after a few days after the coronavirus enters the body with or without symptoms [9]. Other people have severe signs and symptoms of the disease, including involvement of the respiratory system and specifically the lungs [8]. Lung involvement can continue until complete lung destruction and even death of the patient [10]. Epidemiological studies in patients with Covid 19 show that heart disease may be one of the predictors of severe complications in these patients [11]. The results of a study of 41 patients with severe coronary symptoms in Wuhan showed that 6 out of 41 patients (15%) had coronary artery disease [7]. The results of a meta-analysis based on the findings of 6 studies and a total of 1527 patients, showed that 16.5% of patients with Covid 19 were patients with cardiovascular and cerebrovascular diseases [11]. It even seems that patients who suffer a heart attack due to coronary artery disease may have difficulty diagnosing and treating the disease promptly during the COVID-19 epidemic [12]. In many resources, it was argued that the effect of COVID-19 on the patency rate of coronary artery bypass grafts [13].

Considering the high number of patients with coronary artery disease and the large number of coronary artery bypass surgeries in Iran, no study was found that accurately examines the status of the COVID-19 virus in patients undergoing coronary artery bypass surgery. It seems that paying attention to this issue can help healthcare professionals how to control the condition and deal with patients after coronary artery bypass surgery in the condition of COVID-19. Therefore, this study was conducted to determine the frequency of symptoms and signs of COVID-19 in patients who underwent CABG. Of course, reporting the signs and symptoms of COVID-19 does not mean a definite confirmation of this disease, but considering the current epidemic, it can be acknowledged that the reported symptoms were related to COVID-19. Due to the public awareness of the disease of COVID-19, it may be that the reporting of signs and symptoms is consistent with the disease.

## Materials and methods

This study is a descriptive and cross-sectional study performed in an educational therapeutic cardiac referral center in Rasht in northern Iran. The population of this research was made up of patients who had undergone CABG within two years before conducting the research. The inclusion criteria consisted of performing coronary artery bypass graft surgery in the past two years, having the ability to answer questionnaire questions over the phone, and giving verbal consent to participate in the research. The criteria for not entering were not answering the phone call and not agreeing to answer all the questions. Considering that the official announcement of the start of the COVID-19 epidemic in Iran was on February 20, 2020, sampling was done on patients who had undergone CABG during the two years before the start of the COVID-19 epidemic. In this way, the patients who underwent CABG two years before the beginning of the epidemic, between February 2018 and February 2020, were included in the research.

The research instrument consisted of two parts. In the first part, individual characteristics [age, sex, place of residence, living conditions (alone, with spouse, with spouse and children, with children, with others), history of hospitalization after CABG, smoking history after CABG] and variables related to the disease [ See a physician after the onset of Covid 19 symptoms, hospitalization due to Covid 19 symptoms, duration of hospitalization due to Covid 19 disease, home quarantine, attending patient care convalescent after hospital discharge, decreased sense of smell, decreased sense of taste, corona PCR test, positive PCR test, lung scan, diabetes history, hypertension history and hyperlipidemia history ]were questioned. The second part of the 12-item screening checklist was to get the signs and symptoms of Covid 19 with a minimum score of zero and a maximum of 20, and earning a score of at least 3 signs was a significant suspicion of getting Covid 19. Items listed in the tool included the following: history of face-to-face contact with a positive Covid patient within 2 weeks before the onset of symptoms, Temperatures greater than 37.8 degrees measured by mouth, sore throat or severe dryness in the throat, dry cough, diffuse muscle pain, clear runny nose or frequent sneezing, headache, nausea and vomiting, diarrhea, pain or heaviness in the chest area, shortness of breath or respiratory distress and Arterial blood oxygen saturation less than 93%. The answers were designed to say yes/no. This checklist was researcher-made and based on limited pandemic time resources. After designing based on the sources, it was reviewed by seven anesthesiologists and nurses specializing in the care of patients admitted to intensive care units, and according to their opinions, the necessary corrections were made. Due to the nature of the questions, there was no need to determine reliability.

Data were collected by census method by one of the research teams. Since the research was conducted in the spring of 2020, at the height of the COVID-19 epidemic crisis, it was not possible to collect data face-to-face due to general quarantine. Therefore, after confirming the title of the research in the university ethics committee, the telephone number registered in the hospital information system was used to contact those who had undergone CABG. After the approval of the ethics committee (code: IR.GUMS.REC.1399.090), data collection was done. The time to receive the official license for research and data collection was May 28, 2020. Data collection was performed during the last three weeks of June and the first week of July in 2020. Quarantine regulations were in force in Iran at that time. Schools and universities were closed, and organizations were willing to provide services with a minimum workforce and provided with minimal presence and using the Internet. All telephone calls were made from ten to noon Data were analyzed using descriptive statistics, Fisher’s exact test, Mann Whitney U test, and logistic regression model.

## Results

Out of 296 numbers registered in the archives of the hospital information system of the research site, only 200 answered their phones (response rate: 67.56%)0.21 numbers were incorrectly registered, 11 people who had undergone surgery in the past two years had died, 16 people refused to cooperate and did not respond and in other cases, the phones were turned off or the call was not answered. The data obtained from 200 samples and results showed that 10% of the samples had signs and symptoms of Covid 19, according to the instrument. According to the results, the PCR test of these patients was positive and after seeing a physician, their lungs were scanned following the onset of signs and symptoms of Covid 19. Demographic and disease-related characteristics of research samples are listed in Table 1. According to the information in this table, having the symptoms of COVID-19 is significant in terms of the variables of decreased sense of smell, decreased sense of taste, and home quarantine. Then, the logistic regression model was used to determine the most important predictors of Covid signs and symptoms in patients after CABG. The results of the regression model with the backward LR method are shown in Table 2. The model was run in four steps after entering variables with a significant level greater than 0.25. Based on the regression model results, only the variables of living conditions, decreased sense of taste, and lack of home quarantine remained in the final model. Among these variables, only two variables decreased sense of taste and no home quarantine during the Covid epidemic, predicted the onset of Covid signs and symptoms in patients with CABG history.

**Table 1:** Demographic and disease-related characteristics of samples with and without signs and symptoms of Covid-19

Variables		With signs and symptoms of Covid n(%)	Without signs and symptoms of Covid n(%)	Total n(%)	Sig.
Sex	Female	7(3.5)	59(29.5)	66(33)	0.8*
	Male	13(6.5)	121(60.5)	134(67)	
Age	≤ 65	14(7)	114(57)	128(64)	0.3**
	> 65	6(3)	66(33)	72(36)	
Place of residence	City	15(7.5)	117(58.5)	132(66)	0.8*
	Village	5(2.5)	63(31.5)	68(34)	
Living conditions	Alone	0(0)	14(7)	14(7)	0.2*
	With spouse	8(4)	45(22.5)	53(26.5)	
	With spouse and children	12(1.5)	121(60.5)	133(66.5)	
History of hospitalization after CABG	Yes	2(1)	11(5.5)	13(6.5)	0.6*
	No	18(9)	169(84.5)	187(93.5)	
Smoking history after CABG	Yes	5(2.5)	37(18.5)	42(21)	0.5*
	No	15(7.5)	143(71.5)	158(79)	
See a physician after the onset of Covid 19 symptoms	Yes	2(1)	3(1.5)	5(2.5)	0.07*
	No	18(9)	177(88.5)	195(97.5)	
Hospitalization due to Covid 19 symptoms	Yes	1(0.5)	0(0)	1(0.5)	0.1*
	No	19(9.5)	180(90)	199(99.5)	
Home quarantine	Yes	17(8.5)	177(88.5)	194(97)	0.01*
	No	3(1.5)	3(1.5)	6(3)	
Attending patient care convalescent after hospital discharge	Yes	0(0)	0(0)	0(0)	-
	No	20(10)	180(9)	200(100)	
Decreased sense of smell	Yes	5(2.5)	6(3)	11(5.5)	0.002*
	No	15(7.5)	174(87)	189(94.5)	
Decreased sense of taste	Yes	5(2.5)	6(3)	11(5.5)	0.002*
	No	15(7.5)	174(87)	189(94.5)	
	No	12(6)	168(84)	180(90)	
Diabetes history	Yes	13(6.5)	83 (41.5)	96 (48)	0.1*
	No	7(3.5)	97 (48.5)	104 (52)	
Hypertension history	Yes	11(5.5)	89(44.4)	100(50)	0.8*
	No	9(4.5)	91(45.5)	100(50)	
Hyperlipidemia history	Yes	11(5.5)	78(39)	89(44.5)	0.3*
	No	9(4.5)	102(51)	111(55.5)	

*Fisher`s exact test

**Mann Whitney U test

**Table 2:** Results of Logistic regression analysis to predict risk factors related signs and symptoms of Covid 19 after CABG

Variable	B	S.E.	Wald	df	Sig.	Exp(B)	95%CI for Exp(B)		
							Lower	Upper	
Living conditions			2.775335	3	0.506				
Living conditions(alone)	-19.76	9925.7	0.0001	1	0.998	0.0001			
Living conditions (with spouse)	0.51	1.105	0.214	1	0.644	1.667	0.191		14.521
Living conditions (with spouse and children)	-0.313	1.079	0.084	1	0.772	0.731	0.088		6.057
Decreased sense of taste versus no decreased sense of taste	1.942	0.744	6.806	1	0.009	6.071	1.621		29.984
Being in home quarantine versus no quarantine	-2.80	1.278	4.819	1	0.028	0.061	0.005		0.741
Constant	0.352	1.578	0.050	1	0.824	1.421			

## Discussion

This study aimed to determine the frequency of cases of Covid 19 in patients with a history of surgery. Since the data collection of this research was done by phone and a checklist was used to determine the signs and symptoms, The results showed that patients who were likely to develop COVID-19 based on the checklist in question were also tested by PCR to detection Covid and PCR test was positive in 6 out of 20 cases that had signs and symptoms of Covid. According to a study by Wang et al. In China, which examined 300 patients after CABG between January and June 2020, not a single case of Covid 19 was reported [14]. It should be noted, that the study by Wang et al. was performed on patients who had undergone CABG two weeks ago, while the present study examined all patients who underwent CABG before December 2019 up to three years earlier, before the announcement of the COVID-19 pandemic in Iran on February 20, 2020. However, some researchers believe that Current evidence is limited for the frequency of patients with COVID-19 after CABG [15, 16]. Some researchers have suggested the use of telemedicine in the context of the COVID-19 epidemic to monitor the condition of patients after surgery [17–19]. Unfortunately, despite numerous studies on the prevalence of severe complications of Covid 19 in patients with heart disease [20–22], there are very few studies that have examined exclusively patients undergoing surgery. The results of a study by Shakiba et al., Which was performed on 528 people in northern Iran using a serological test, revealed a 22.2% incidence of COVID-19 [23].

The results of the present study showed that the frequency of Covid 19 symptoms was significant based on home quarantine, reduced sense of smell, and decreased sense of taste. European reports on coronavirus disease 19 (COVID-19) patients are detecting a frequency of these symptoms between 19.4% and 88% [24–28]. According to the results of several studies, a decreased sense of taste and smell is one of the symptoms of Covid 19, in the majority of people who had the symptoms of COVID-19 in the present study, decreased sense of smell and taste. Has also been reported. The results of Kosugi et al. s study showed that the COVID test was positive in 79.2% of 253 patients with olfactory disorders [29]. According to the results of this study, the occurrence of signs and symptoms of COVID-19 was significant in terms of home quarantine. Since the announcement of the COVID-19 epidemic in Iran, in the city of data collection, Rasht in northern Iran, people have been asked to observe home quarantine and leave home only to do essential work. Those who had a sign or symptom of Covid 19 seem to have adhered to this protocol for fear of severe complications.

The use of a regression model showed that the variables of decreased sense of taste and non-adherence to home quarantine were predictive variables of signs and symptoms of Covid 19. Thus, those who had a history of CABG were more likely to develop Covid 19 symptoms if their sense of taste was reduced and home quarantine was not observed. In a study conducted on 1480 Iranian citizens in 2020, more than 93% of the surveyed units were aware of the usefulness of avoiding crowded places and the need to be at home [30]. Given that all participants in the present study had a history of CABG, and that they or their caregivers were probably sufficiently aware of the risk of developing the disease after exposure to other individuals Also the majority of people who did not have the signs and symptoms of Covid 19 in this study were quarantined at home. It seems the participants in this study knew in an epidemic, the chances of encountering seemingly healthy disease carriers increased, so they quarantined themselves at home.

We have encountered some limitations in this study. First, due to the COVID-19 epidemic and the high-risk research community, face-to-face interviews were not possible. Telephone data collection may not reflect all of reality. Second, the study was retrospective and there may be a recall error. At the same time, it was not possible to perform a test for COVID-19 and the results were analyzed based on the response to the checklist of signs and symptoms. However, given the public awareness of the signs and symptoms of the disease, it can be hoped that if there was no possibility of memory errors, the results would be closer to reality. In addition in the research center, according to the medical records, access has been available for the past three years and the response rate was 67.56%. It was not possible to access the rest of the people who underwent CABG, and different results may have been obtained if they had responded.

Based on the findings of the study and its limitations, it is suggested that the frequency of COVID-19 infection in people who have undergone CABG be studied based on the registered medical records of individuals who had a definitive diagnosis of Covid 19. Given that many aspects of Covid disease remain unknown and that universal vaccination is not possible in many countries, identifying predictors of increased risk of disease can lead to more accurate care protocols.

## Conclusion

The results did not indicate the frequency of COVID signs and symptoms among people with a history of coronary artery bypass surgery more than healthy people in the Iranian community. Extensive studies are suggested in this regard. It should be noted that after coronary artery bypass surgery, coronary artery disease is not definitively cured and only the rate of progression of coronary disease signs and symptoms decreases. Therefore, there is an underlying disease in these patients until the end of life. Unfortunately, in addition to reducing or disrupting the blood supply to the heart tissue, cardiovascular disease can be accompanied by a decrease in blood supply to all body organs in the long term. In this case, the ability to fight against many infections, including the COVID-19 virus, will probably decrease by affecting the immune system. It seems that during the outbreak of viral diseases such as COVID-19, for which there is no definitive treatment, it is more important to observe caution for patients with coronary artery disease - even after coronary artery transplant surgery. Coronary artery bypass surgery can prevent the development of acute signs and symptoms of cardiac tissue ischemia. Still, due to the stability of the factors that were the main cause of coronary artery stenosis and cardiac ischemia, such as high blood pressure, suffering from diabetes, inappropriate lifestyle, and not following medication and care instructions, there is a possibility of the disease progressing. Therefore, it cannot be assumed that the patient with cardiac ischemia and blood supply disorder has fully recovered after the coronary artery transplant surgery. Therefore, it is very likely that the ability of their coping systems cannot be considered similar to peers who do not suffer from cardiovascular disease. It should be noted that this study was conducted only on 200 samples, using a questionnaire, without conducting necessary examinations and checks, and using telephone calls, according to the conditions during the COVID-19 epidemic. About one-third of the people who had undergone coronary artery bypass surgery within two years before the data collection of this study did not participate in the research for various reasons, which could have affected the results of the research. The samples were available and they entered the research non-randomly. All these things can affect the results of the research. Of course, it should be noted that in some seasons of the year and some regions of the world, there are cases of infection with different strains of the coronavirus, so it is suggested to conduct studies with a cross-sectional design on the frequency of infection with these strains in patients who are suffering from cardiovascular diseases or have undergone coronary artery transplant surgery during the outbreak of coronavirus strains. Of course, due to the implementation of the universal vaccination program against the COVID-19 virus, some studies can be done considering the effects of vaccination history. The physiopathology of the impact of the COVID-19 virus on the human body has not been accurately identified, there is a possibility that more destructive effects will be seen in patients with coronary artery disease. It should be noted that the joint function of the heart and lungs causes any heart damage to affect the respiratory function of patients, and considering the stability of the complications of coronary artery disease in these patients, despite performing surgery, taking care to prevent viral infections and especially Covid-19 seems necessary. It seems that preventive care should be considered more seriously in patients undergoing coronary artery bypass surgery. Teaching self-care and avoiding exposure to viral infections can help in maintaining the health of these patients.

## Data Availability

The datasets used and analyzed during the current study available from the corresponding author on reasonable request.
